# Integrated Transcriptome and Histone Modification Analysis Reveals NDV Infection Under Heat Stress Affects Bursa Development and Proliferation in Susceptible Chicken Line

**DOI:** 10.3389/fgene.2020.567812

**Published:** 2020-09-25

**Authors:** Ganrea Chanthavixay, Colin Kern, Ying Wang, Perot Saelao, Susan J. Lamont, Rodrigo A. Gallardo, Gonzalo Rincon, Huaijun Zhou

**Affiliations:** ^1^Department of Animal Science, University of California, Davis, Davis, CA, United States; ^2^Department of Animal Science, Iowa State University, Ames, IA, United States; ^3^School of Veterinary Medicine, University of California, Davis, Davis, CA, United States; ^4^Zoetis Inc., Parsippany, NJ, United States

**Keywords:** Newcastle disease virus, heat stress, bursa, RNA-seq, ChIP-seq

## Abstract

Two environmental factors, Newcastle disease and heat stress, are concurrently negatively impacting poultry worldwide and warrant greater attention into developing genetic resistance within chickens. Using two genetically distinct and highly inbred layer lines, Fayoumi and Leghorn, we explored how different genetic backgrounds affect the bursal response to a treatment of simultaneous Newcastle disease virus (NDV) infection at 6 days postinfection (dpi) while under chronic heat stress. The bursa is a primary lymphoid organ within birds and is crucial for the development of B cells. We performed RNA-seq and ChIP-seq targeting histone modifications on bursa tissue. Differential gene expression revealed that Leghorn, compared to Fayoumi, had significant down-regulation in genes involved in cell proliferation, cell cycle, and cell division. Interestingly, we also found greater differences in histone modification levels in response to treatment in Leghorns than Fayoumis, and biological processes enriched in associated target genes of H3K27ac and H3K4me1 were similarly associated with cell cycle and receptor signaling of lymphocytes. Lastly, we found candidate variants between the two genetic lines within exons of differentially expressed genes and regulatory elements with differential histone modification enrichment between the lines, which provides a strong foundation for understanding the effects of genetic variation on NDV resistance under heat stress. This study provides further understanding of the cellular mechanisms affected by NDV infection under heat stress in chicken bursa and identified potential genes and regulatory regions that may be targets for developing genetic resistance within chickens.

## Introduction

Infection with the paramyxovirus, Newcastle disease virus (NDV), in chickens has resulted in devastating economic losses across both commercial and backyard poultry farms with mortality rates as high as 100% (Alexander, 2000). The most effective method in preventing and controlling the spread of Newcastle disease (ND) is through biosecurity and vaccines, which when administered effectively can provide up to 100% survival against lethal, velogenic NDV infection in chickens (Stone et al., 1980; Miller et al., 2013). However, vaccination is not a practical option for many farmers in developing countries because vaccination programs are costly, have limited support, and lack proper infrastructure. An additional protective measure that has not been extensively explored is the use of genomics and genetics to improve NDV resistance. Therefore, our efforts have been in identifying genetic variation that is associated with NDV resistance as a method for mitigating the potentially devastating effects of virulent NDV. Variation in NDV resistance has previously been observed between different breeds and lines that were first observed during previous outbreaks (Albiston and Gorrie, 1942).

With global climate change occurring, there is increasing concern about the effects of heat stress. Poultry are experiencing more frequent events of higher temperatures and associated humidity levels, which are likely coupled with detrimental effects of NDV infection. Heat stress is well-known to have negative effects on economic traits such as decreased meat and egg quality and quantity, decreased reproduction, and, in extreme cases, increased mortality (St-Pierre et al., 2003; Mashaly et al., 2004; Barrett et al., 2019; Zaboli et al., 2019). More relevant to our concerns are the effects of heat stress on the immune system. Several studies in chickens under heat stress have provided evidence of increased cell-mediated immunity by stimulating greater numbers of circulating heterophils, which are the predominant leukocyte within birds (Maxwell et al., 1992; Prieto and Campo, 2010). The negative effects of heat stress appear to predominantly be in the humoral and adaptive arm of the immune system. Several studies have shown that heat stress in chickens caused a reduction of antibody levels following vaccination and decreased total white blood cells (Thaxton et al., 1968; Nathan et al., 1976; Maxwell et al., 1992). These results suggest that there is a negative impact that can affect vaccine efficacy or the host’s ability to fight infection because B cells are the host’s antibody-producing cells and play a large role in building protection against NDV.

The bursa of Fabricius is an organ vital in the development of B cells in avian species. B cells are responsible for producing the host’s natural and antigen-specific antibodies, conferring general and broad protection during primary infection and pathogen-specific protection for recurring infections. This essential function of B cells relies on the bursa as a primary lymphoid organ that provides the only site where gene conversion occurs for immunoglobulin (Ig) gene diversification that is critical for producing the B-cell repertoire. At hatching, the bursa is composed almost entirely of B cells (Ko et al., 2018) and continues growing in weight to peak between 5 and 8 weeks posthatching after which involution begins until the organ completely disappears around the age of sexual maturity (∼20–25 weeks) (Ratcliffe and Härtle, 2014). Bursectomy in 2-week-old or younger chicks resulted in reduced hemagglutination inhibition antibody formation to NDV compared to control chicks, illustrating that the bursa plays a significant role in protection against NDV (Matsuda and Bito, 1973). Therefore, exploring the bursal response to NDV infection under heat stress is important for understanding what responses contribute to host resistance.

One of the most important conclusions drawn from reviews of genome-wide association studies (GWAS) is that the majority of trait-associated variants lie within non-coding regions of the genome (Hindorff et al., 2009; Maurano et al., 2012). Even the few studies that performed GWAS for NDV resistance found that most associated single-nucleotide polymorphisms (SNPs) and quantitative trait loci (QTLs) lie within or overlap with non-coding regions (Yonash et al., 2001; Biscarini et al., 2010; Luo et al., 2013; Saelao et al., 2019). Therefore, identification and annotation of genome-wide non-coding regions will aid in identifying regulatory elements and putative causative variants possibly associated with NDV resistance. Here, we performed chromatin immunoprecipitation followed by high-throughput sequencing (ChIP-seq) of histone modifications that have known associations with genome regulatory elements and their state of activities. We selected two active histone modifications: H3K4me3 and H3K27ac, with H3K4me3 predominantly marking active promoters and H3K27ac marking both active promoters and enhancers (Guenther et al., 2007; Mikkelsen et al., 2007; Nègre et al., 2011). H3K4me1 also marks both promoters and enhancers but is not strongly associated with activity and, when appearing on enhancers in the absence of H3K27ac, indicates a poised or primed enhancer (Creyghton et al., 2010; Ernst et al., 2011). H3K27me3 is associated with polycomb-mediated repression, spanning over large repressed regions or regulatory elements (Cao et al., 2002; ENCODE Project Consortium, Birney et al., 2007). To extend our exploration of genome-wide changes within functional genomic regions, transcriptome analysis from RNA-seq was utilized to identify differential gene expression associated with treatment or genetic line in order to identify genes and biological pathways contributing to treatment responses.

The aims of this study were to annotate regulatory elements in chicken bursa and identify genes, regulatory elements, and biological processes of the bursal response contributing to host resistance to NDV infection under heat stress. We took advantage of two genetically distinct and inbred chicken lines, Fayoumi and Leghorn, with Fayoumi being the more relatively resistant line compared to Leghorn as observed through their lower NDV titers and higher NDV-specific antibody levels. Additionally, Fayoumi is hypothesized to be more heat stress–resistant because the line originated from Egypt therefore historically had adapted through natural selection to an environment with higher climate (Wang et al., 2014; Deist et al., 2017; Saelao et al., 2018b). Physiological responses to heat stress have been shown to be significantly different between the two lines, with electrolyte levels appearing to be more well-maintained during heat stress in Fayoumis compared to Leghorns (Wang et al., 2018).

## Materials and Methods

### Experimental Design

This study is part of a collaborative project for the Feed the Future Innovation Lab for Genomics to Improve Poultry program^1^. The project involves studying the host genetic contribution to NDV resistance alone and NDV resistance under heat stress, with the latter being the focus of our study and the former at Iowa State University (ISU). The two inbred chicken lines used in this study are Fayoumi (M15.2) and Leghorn (GHs 6), which are maintained at ISU (Ames, IA, United States). The experimental design has been described in previous publications (Saelao et al., 2018a, b; Wang et al., 2018). Upon arriving from ISU, chicks were randomly divided into a non-treated and treated group for each line. Both groups were reared at 29.4°C and 60% humidity until 13 days of age after which the treated group at 14 days of age was subjected to heat stress. Heat stress treatment consisted of 38°C for the first 4 h, after which decreased to 35°C for the remainder of the trial. At 21 days of age, the treated group, while still under heat stress, was inoculated with a total of 200 μL 10^7^ EID_50_ through oral and nasal passages. The non-treated group (25 birds each line) was kept at 25°C and inoculated with phosphate-buffered saline (PBS) at 21 days of age. Four randomly selected birds at each time point were euthanized by CO_2_. Bursa tissue collected at 6 days postinfection (dpi) was snap-frozen and submerged in RNA*later* for storage in 80°C (Thermo Fisher, Waltham, MA, United States #AM7024). The experiment’s procedures were performed according to the guidelines approved by the Institutional Animal Care and Use Committee at the University of California, Davis (IACUC #17853).

### RNA-Seq Experiments

Total RNA (four individuals per group) from bursa tissue stored in RNA*later* was extracted using TRIzol (Thermo Fisher, #15596026), treated with DNase, and purified through ethanol precipitation. Total RNA for each sample was sent to Novogene Co., Ltd. (Beijing, China) where RNA integrity number determined using an Agilent’sBioanalyzer (Agilent Technologies, Santa Clara, CA, United States) was confirmed to be 7 or above. Then, non-directional RNA-seq libraries were prepared and sequenced using Illumina’s Hiseq 4000 (Illumina, San Diego, CA, United States) with paired-end 150–base pair (bp) reads.

### RNA-Seq Analysis

Adaptors were trimmed and low-quality reads removed using Trim Galore! (Krueger, 2012) version 0.4.5, with default parameters and aligned using STAR (Dobin et al., 2012) version 2.7.2a to the chicken galGal6 genome assembly and Ensembl annotation 95. Reads were filtered based on alignment quality using SAMtools (Li et al., 2009) version 1.9. Supplementary Table 1 shows a summary of read alignment for each sample. Raw read counts were obtained using HTSeq (Anders et al., 2014) version 0.9.1, and differential gene analysis was performed with DESeq2 (Love et al., 2014) version 1.22.2 with a design that included treatment group, genetic line, and batch group that refers to the sample prep. Transcript per million expression values were obtained by using StringTie (Pertea et al., 2015) version 2.0.4.

### ChIP-Seq Experiments

ChIP-seq experiments (two biological replicates per group) were performed on 20–30 mg of frozen tissue per sample using the iDeal ChIP-seq kit for Histones (#C01010059) (Diagenode Inc., Denville, NJ, United States). Tissue was homogenized using liquid nitrogen with mortar pestle and then transferred to 1.5-mL microcentrifuge tube containing lysis buffer iL1 from the kit. Cross-linking was performed for 8 min using 1% formaldehyde that was produced from Pierce 16% methanol-free formaldehyde (Thermo Fisher, #28906), quenched with glycine for 10 min, washed twice with PBS, and resuspended in iL1 lysis buffer. After incubating for 20 min on ice, tissue was homogenized with a Dounce homogenizer, centrifuged 5 min at 2,000 × *g*, and resuspended in iL2 buffer for incubation on ice for 10 min. Nuclei were harvested by centrifugation at 2,000 × *g* for 5 min and resuspended in iS1 buffer for incubation on ice for 30 min. All centrifugation steps were performed at 4°C. Chromatin was sheared using the Covaris E220 in snap-cap microTUBEs (Covaris, Inc., Woburn, MA, United States, #520077) for 6 min at 105 W and then 6 min at 140 W. For immunoprecipitation experiments, about 1,000 ng of sheared chromatin (estimated from DNA extraction) was used as input after which the kit protocol was followed with 1 μg of antibody. The antibodies used were from Diagenode Inc.: H3K4me3 (provided in kit), H3K27me3 (#C15410069), H3K27ac (#C15410174), and H3K4me1 (#C15410037). Each sample had ChIP performed for the four histone modifications and an input (no antibody). Libraries were prepared using the NEBNext Ultra DNA library prep kit for Illumina (New England Biolabs, Ipswich, MA, United States, #E7645L), selecting for 150- to 200-bp insert fragment sizes. Libraries were sequenced on Illumina’s HiSeq 4000 with single-end 50-bp reads.

### ChIP-Seq Analysis

Adaptors were trimmed and low-quality reads removed using Trim Galore! version 0.4.5 and aligned using BWA (Li and Durbin, 2009) version 0.7.17 to the chicken galGal6 genome. Duplicate reads were marked and removed using SAMtools version 1.9 to eliminate PCR amplification bias. Bam files were converted to tagalign files using BEDtools (Quinlan and Hall, 2010) (version 2.27.1) bamtobed command, which were then input files into MACS2 (Zhang et al., 2008) version 2.1.1 peak caller. For all samples, peak calling included the parameters –to-large and –fix-bimodal. For narrow peaks, which included all modifications except H3K27me3, the cutoff for significance was *q* < 0.01 and included the parameter –call-summits. For broad peaks, the cutoff for significance was *q* < 0.05 and included the parameter –broad. The Jensen–Shannon distance (JSD) quality metric was calculated from the PlotFingerprint command from deepTools 2.0 version 3.3.0 and essentially is a measurement comparing mark sample versus input sample. Measurements should be between 0 and 1, where a higher value indicates a higher-quality library. Pearson correlation plots were produced using deepTools (Supplementary Figure 1). Final read count, number of peak calls, genome coverage, and quality metrics for all individual samples and number of peak calls for each combined group are summarized in Supplementary Table 2. Based on the measurements above, we observed that one of the treated Fayoumi samples (1107) appears to be an outlier. Therefore, we removed sample 1107 from further analysis. Combined group peak calls were identified by combining peak calls from both replicates within each group. Peaks that were called in only one replicate but were found to have enrichment in the second replicate were also retained in the combined group peak calls. Pipelines used in this study for peak calling are available at https://github.com/kernco.

### Chromatin State Prediction From ChromHMM

Combined group peak calls were used as the input files for ChromHMM (Ernst and Kellis, 2012) to build a model of state annotations based on the genome-wide combinatorial patterns of the histone modifications across all samples. The most optimal number of chromatin states was determined by creating models between 10 and 16 states using data from all samples and then using only samples within each of the four biological groups. For each state model between 10 and 16, a correlation was calculated for each state between the all-sample model to each of the smaller models created for each of the four groups. An average correlation value was calculated for each state model, and the highest correlation was observed for the 13-state model. The 13-state model was annotated into regulatory regions and their state of activity using several criteria: enrichment around the transcription state site (TSS) and median gene expression as shown in Supplementary Figure 2, the known association of each histone modification to regulatory elements and activity prediction as previously described in the introduction, and examples from previously annotated chromatin state modes (Ernst et al., 2011; Roadmap Epigenomics Consortium, Kundaje et al., 2015). In order to assign the promoter regions a chromatin state, a 4,000-bp region centered on the TSS was annotated with the most prevalent overlapping state excluding the low signal/heterochromatin state (state 10). However, the promoter was reassigned to the following states if there was any overlap within the region, which are also ordered by priority if more than two of these occurred: active TSS (state 6), Poised TSS (state 8), and then repressed TSS (state 13).

### Determining Regulatory Regions for Enrichment Analysis

Given the challenge in how to integrate multiple histone marks to determine the regulatory regions that were used to calculate the enrichment levels, we utilized the chromatin state model for this purpose. First, adjacent states were merged using BEDtools (Quinlan and Hall, 2010) version 2.27.1. Then, the co-occurrence of two states were counted and presented as the percentage of a state’s total count number in order to observe which chromatin states have a tendency to co-occur together within the same regulatory regions (Supplementary Figure 3). Only high confidence states that specifically identified promoters and enhancers were considered; therefore, states 7, 10, and 11 were removed. By looking at the co-occurrence of the states, we observed that in general the active states tend to co-occur together in the same regulatory region and *vice versa* for poised/repressed regions. However, there were still instances where active states co-occur with poised states and, to a lesser degree, repressed states. The co-occurrence analysis, along with visualization of chromatin states and histone peaks on the genome browser (Supplementary Figure 4), revealed that multiple chromatin states can characterize one regulatory element and therefore cannot be considered separately for the enrichment analysis when a histone modification peak can overlap multiple chromatin states. For these reasons, adjacent states (excluding states 7, 10, and 11) were merged together, resulting in a total of 45,596 regions that were divided into 15,856 promoter regions and 32,055 distal from promoter regulatory regions (Supplementary Figure 5).

### Histone Modification Enrichment Analysis

Enrichment levels for each histone modification were calculated using deepTools’ (version 3.3.0) multiBamSummary command to count reads overlapping the regulatory regions produced from merged chromatin states. DESeq2 version 1.22.2 was used to determine differentially enriched regions (DERs) between groups. Normalized read counts were used to produce principal components analysis (PCA) plots (Figure 5). Previously, Pearson correlation was used for quality checking ChIP-seq samples because Pearson correlation is more appropriate when comparing samples within a histone modification. Additionally, because the whole genome was utilized to calculate correlation, there will be much more noise that affects the Spearman correlation more than the Pearson correlation. However, when comparing enrichment level fold changes between histone modifications and against gene expression, the measurements are between different assays that may not necessarily be changing at constant rate together, making the Spearman correlation more appropriate for these comparisons.

### Functional Analysis of Differentially Expressed Genes, Regulatory Regions, and Their Associated Target Genes

Associated target genes of DERs were determined by identifying the nearest TSS to each regulatory element. GO terms for biological processes (GO_TERM_BP) and KEGG pathways were identified for differentially expressed genes (DEGs) and associated target genes of DERs using database for annotation, visualization, and integrated discovery (DAVID) (Huang et al., 2007) version 6.8. Ensembl gene IDs were converted to official gene symbols for input into DAVID using Ensembl’s Biomart. Significance cutoff was *p* < 0.05. The background chosen was *Homo sapiens* because terms and pathways are much more improved in annotation than *Gallus gallus*, especially for immune-related processes. Additionally, uninformative pathways related to cancer, which were broad and/or unrelated to the tissue or treatment in the study, were removed from the top 5 pathways shown for the KEGG pathway analysis for Leghorn within-line DEGs. *De novo* transcription factor motif analysis was performed with HOMER (Heinz et al., 2010). The median width of regulatory regions was 2,400 bp; therefore, a 2,400-bp region centered on each DER was used as input for HOMER. Background regions for comparison against the input regions were randomly generated by HOMER.

### Variant Analysis

Whole-genome sequencing data from DNA-seq of Fayoumi and Leghorn birds from Fleming et al. (2016) was used. Adaptors were trimmed and low-quality reads removed using Trim Galore! version 0.4.5 and aligned to the galGal6 reference genome using BWA version 0.7.17. Reads were filtered based on alignment quality (*q* < 30), and duplicate reads were marked with Picard tools were then removed with SAMtools version 1.9. Variant calling of single-nucleotide variants (SNVs) and indels were called using GATK (Van der Auwera et al., 2013) (version 4.1.4.1) HaplotypeCaller command. The ploidy was set to 2, even though the pooled library contained 16 individuals. However, we were only considering homozygous variants between lines; therefore, any heterozygous variants within any individual in a genetic line would have been filtered. Variants were hard-filtered with the following arguments: QD < 2.0, FS > 60.0, SOR > 3.0, MQ < 40.0, MQRankSum < −12.5, and ReadPosRankSum < −8.0. Using BCFtools (Li, 2011) version 1.10.2, variants within each line were filtered if total reads < 20, and BEDtools (Quinlan and Hall, 2010) version 2.27.1 was used to identify variants that overlapped exons from the Ensembl galGal6 annotation and regulatory regions used for the histone modification enrichment in this study.

## Results

### Transcriptome Analysis for Treatment and Line Differences

By performing RNA-seq in four groups (combination of two lines and two conditions), four comparisons were made for differential gene analysis on bursa samples from 6 dpi that included within-line comparisons [treated Fayoumis (TF) vs. control Fayoumis (CF) and treated Leghorn (TL) vs. control Leghorn (LC)] and between-line comparisons (CF vs. CL and TF vs. TL).

**FIGURE 1:**
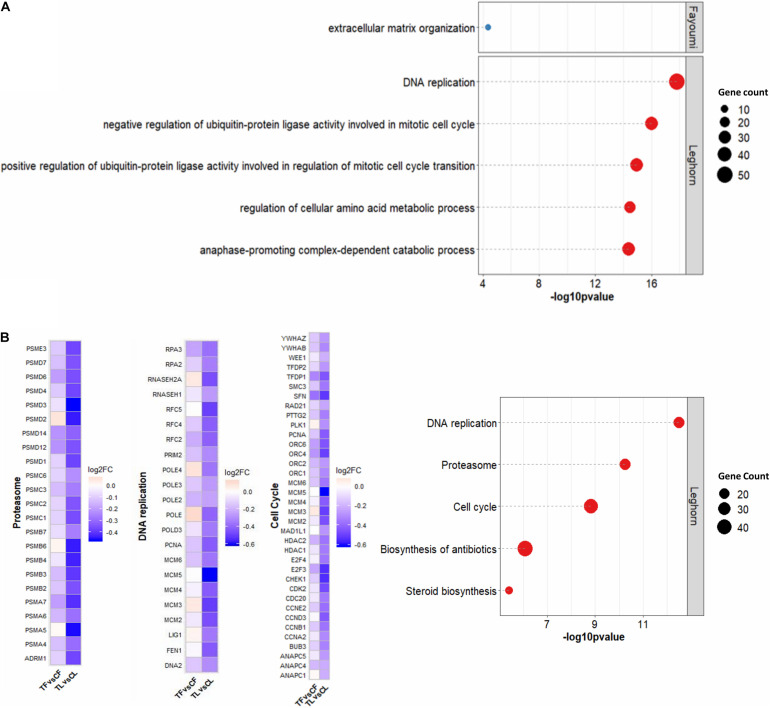
Functional analysis of DEGs for within-line comparisons. **(A)** Top 5 GO terms for biological processes. **(B)** Top 5 KEGG pathways on the right were found for Leghorn only and heat map of the log2 fold changes of the DEGs within the top 3 KEGG pathways related to cell proliferation on the left. The number of genes denotes the number of DEGs associated with the term or pathway. Significant GO terms and KEGG pathway were identified by *p* < 0.05 with more than two gene counts. CF, control Fayoumi; TF, treated Fayoumi; CL, control Leghorn; TL, treated.

Within-line comparisons for Fayoumis and Leghorns identified 54 and 1,592 DEGs, respectively (Supplementary Figure 5A). Interestingly, DEGs identified in Fayoumis were mostly up-regulated, whereas about two-thirds of DEGs in Leghorns were down-regulated. The top 5 GO terms for biological processes and KEGG pathways for DEGs in within-line comparisons are shown in Figures 1A,B. Fayoumis had one significant GO term involving extracellular matrix organization, whereas Leghorns’ top 5 GO terms revealed several pathways that appear to be involved in cell cycle (Figure 1A). Significant KEGG pathways were found only for the within-line comparison of Leghorns (Figure 1B), which also showed pathways related to cell division and cell cycle, as well as other pathways possibly related to heat stress response. In general, considerable down-regulation of DEGs across the top 3 KEGG pathways was observed in the Leghorn line (Figure 1B).

**FIGURE 2:**
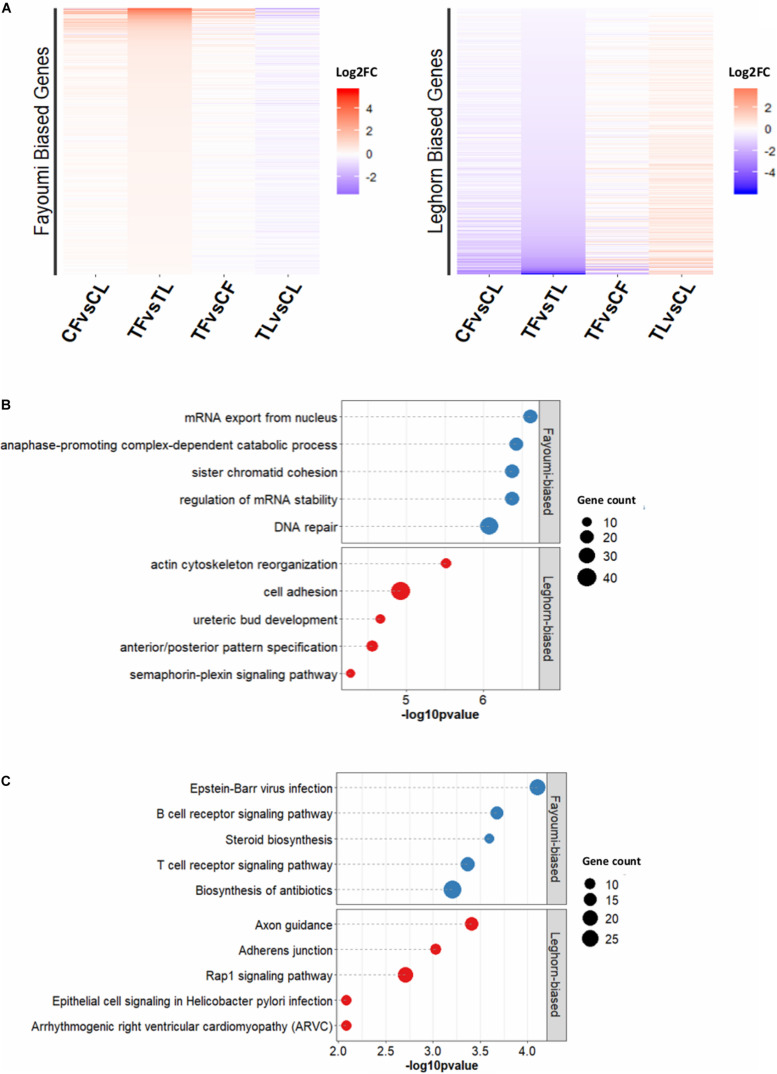
Functional analysis of DEGs specific for treated-line comparison. **(A)** Log2FC for Fayoumi-biased and Leghorn-biased DEGs specifically within treated-line comparison compared to control-line comparison. **(B)** The top 5 GO terms for Fayoumi-biased and Leghorn-biased DEGs specific to the treated-line comparison. **(C)** The top 5 KEGG pathways for Fayoumi-biased and Leghorn-biased DEGs specific to treated-line comparison.

The between-line comparisons identified greater numbers of DEGs regardless of treatment (Supplementary Figure 5B), illustrating that genetic line was a more influential biological factor in gene expression differences compared to treatment. However, the treated-line comparison produced a greater number of DEGs than the non-treated-line comparison, which alludes to the distinct effects of treatment on each line. Focusing on the genetic line differences due to treatment, the 2,524 DEGs that were specific to the comparison between the treated Fayoumi and treated Leghorn (treated-line comparison) were further explored (Supplementary Figure 5C). Separating the DEGs into Fayoumi-biased and Leghorn-biased DEGs (i.e., higher expression in one line), the log2 fold change across all comparisons are presented in Figure 2A, the top 5 GO terms are presented in Figure 2B, and the top 5 KEGG pathways are presented in Figure 2C. Additionally, these DEGs appeared to mainly be due to the changes within Leghorns, where most Fayoumi-biased DEGs or Leghorn-biased DEGs have greater fold changes in expression in the Leghorn within-line comparison (TL vs. CL) compared to the Fayoumi within-line comparison (TF vs. CF) (Figure 2A).

The top 5 GO terms and KEGG pathways for Fayoumi-biased and Leghorn-biased DEGs are shown in Figures 2B,C. In the top GO terms for Fayoumi-biased DEGs, we again observed processes related to cell division with the addition of those related to mRNA processes, whereas for Leghorn-biased DEGs we observed processes related to various cellular and developmental processes. For the top 5 KEGG pathways with Fayoumi-biased DEGs, we found immune-related KEGG pathways including a very relevant pathway, B-cell receptor signaling pathway, to bursa, which is almost entirely comprised of B cells (Weill et al., 1987; Ko et al., 2018). The top KEGG pathway for the Leghorn-biased DEGs was axon guidance with several other pathways related to cellular processes and interactions such as adherens junction and rap1 signaling pathway.

**FIGURE 3:**
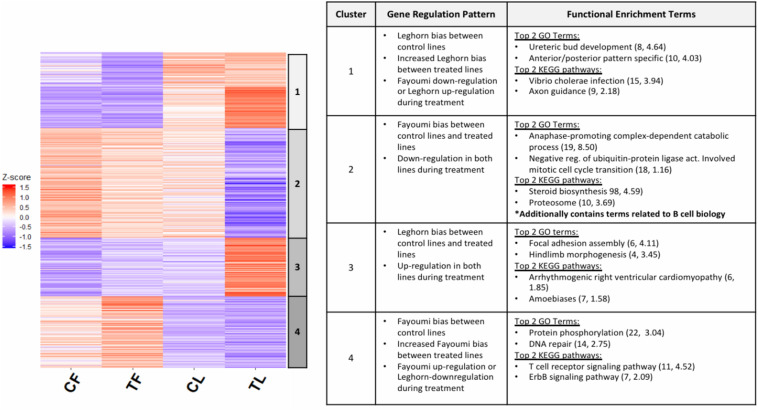
Differentially expressed genes (DEGs) with inherent line biases that increase during treatment show Fayoumi’s ability to retain cell-identity development versus Leghorn. The *z* scores were calculated for DEGs that were specific to the treated-line comparison and used in k-means clustering. Five clusters were chosen for the *k*-means clustering, but two clusters with similar gene regulation patterns were combined into cluster 1, resulting in four clusters shown in the heat map above. The chart summarizes the gene pattern regulation of each cluster that describes whether the DEGs had up-regulation or down-regulation between non-treated and treated birds within each line and whether they were Fayoumi-biased or Leghorn-biased for each between-line comparison. The top 2 GO terms and KEGG pathways are shown for each cluster with the number of DEGs enriched in each pathway and –log10 *p*-value. Significant GO terms and KEGG pathway were identified by *p* < 0.05 with more than two gene counts.

An interesting pattern was observed in the fold changes of the DEGs specific to the treated-line comparison (TF vs. TL, Figure 2A), where a number of those DEGs have a preexisting trend of bias, although not statistically significant (FDR > 0.05), toward one line in the comparison between non-treated lines (CF vs. CL). This suggests that for a set of DEGs that have inherently differential expression between the lines, treatment exacerbates the differences in expression between the lines. Exploring this further, we performed *k*-means clustering on the DEGs specific to the treated-line comparison in order to separate the set of DEGs with this specific pattern described above. Figure 3 shows a heat map of the four clusters with a table that includes descriptions of general regulation patterns observed between lines within a treatment or between treatment groups within a line. The top 2 GO terms and KEGG pathways are also listed for each cluster along with the gene count and -log10 *p*-value. The two clusters that display the specific pattern of interest are clusters 1 and 4. The set of DEGs in cluster 1 show a pattern of Leghorn bias when comparing between non-treated lines (CF vs. CL). This magnitude of Leghorn bias then became increased when comparing treated lines (TF vs. TL). Cluster 4 shows a similar pattern except there is a Fayoumi bias. The biological processes in cluster 1 appeared to be more enriched in early development, whereas in cluster 4 the processes relate to cellular processes and immune signaling of lymphocytes. These results suggest that Fayoumis compared to Leghorns may have a more progressed immune cell identity of the bursa that is preserved during treatment.

### Chromatin State Annotation at Promoters of DEGs

To explore the changes in the activation of *cis*-regulatory regions such as promoters and enhancers due to treatment or genetic line differences, we performed replicate ChIP-seq assays for each group in the following four histone modifications: H3K4me3, H3K27ac, H3K4me1, and H3K27me3. Pearson correlation values between the samples showed replicates within groups are highly correlated and clustered by genetic line and treatment (Supplementary Figure 1). A summary of the number of peaks called and genome coverage of peaks are provided in Supplementary Table 2, alongside the number of aligned and filtered reads and quality metric scores that include FRIP (fraction of reads in peaks) and JSD.

**FIGURE 4:**
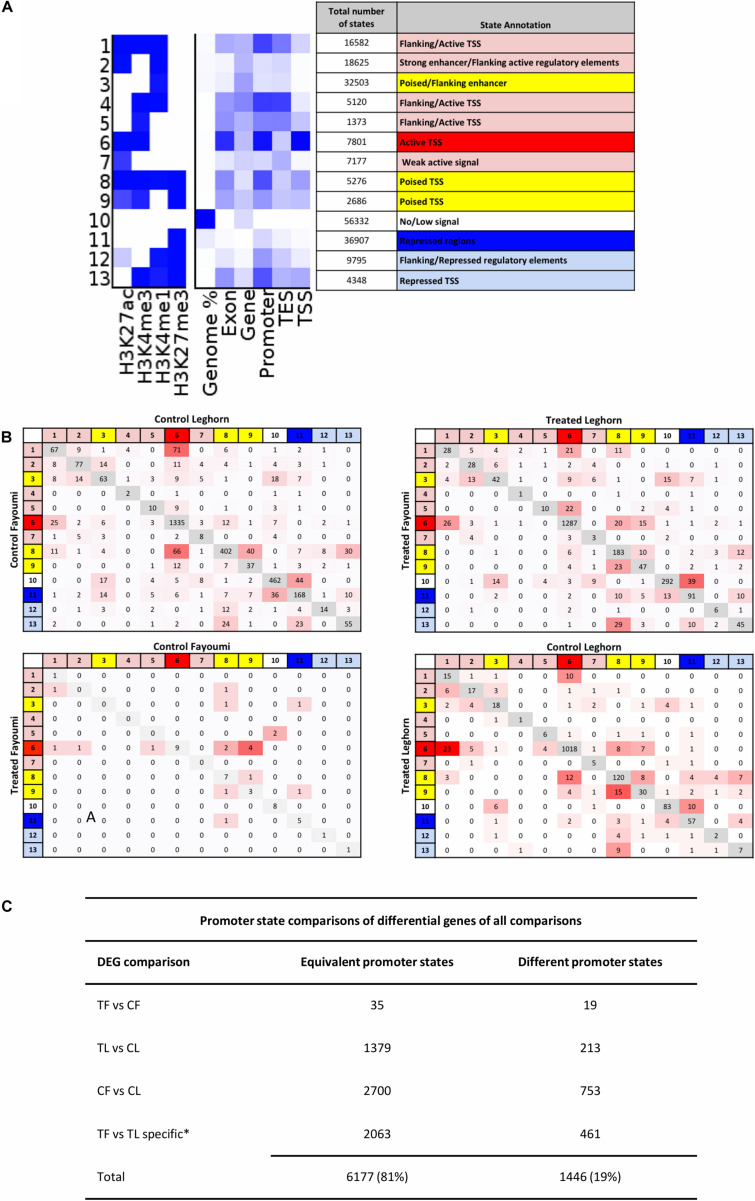
The promoter states of differentially expressed genes generally are not different between groups, suggesting enhancers may play a role in modulating expression for these DEGs. **(A)** Annotation of chromatin states are based on promoter enrichment, gene expression correlation, and known associations of histone modifications to regulatory elements and their activity. States are grouped into three main activity groups: active (red), poised (yellow), and repressed (blue). **(B)** Promoter transition tables are shown for the DEGs in each comparison. **(C)** Table summarizing the states that remain the same between two groups or are different between groups is shown in **(A)** and includes the total number of promoters that were the same and were different between two groups. *Numbers shown for TF versus TL comparison refer to DEGs specific to that gene set in comparison to the CF vs. CL gene set. CF, control Fayoumi; TF, treated Fayoumi; CL, control Leghorn; TL, treated Leghorn.

In order to identify and annotate genome-wide *cis*-regulatory elements, we utilized a tool called ChromHMM. The tool allows integration of multiple histone modifications, which increases confidence in more accurate chromatin state annotation, to identify combinatorial patterns that characterize regulatory regions and the state of activity of regulatory elements for each biological group, thereby facilitating more convenient downstream comparisons of regulatory regions between groups (ENCODE Project Consortium, Birney et al., 2007; Ernst et al., 2011; Roadmap Epigenomics Consortium, Kundaje et al., 2015). A 13-state model was chosen based on criteria explained in Section “Materials and Methods,” and the annotation is shown in Figure 4A. All 13 states were individually annotated but can generally be categorized into active (red), poised (yellow), and repressed (blue) regulatory elements or regions.

**FIGURE 5:**
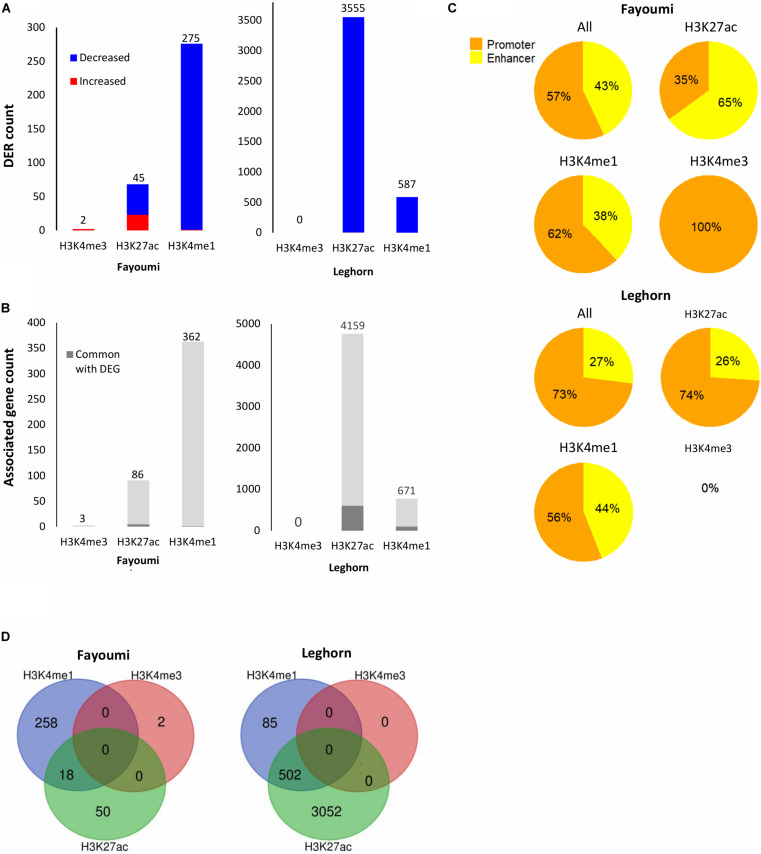
Summary of DER analysis for within-line comparisons. **(A)** Stacked bar plots showing the number of DERs with increased or decreased enrichment identified for each within-line comparison across all histone modifications. **(B)** Stacked bar plots showing the number of unique target genes associated with DERs and the number of associated genes that are in common with DEGs of the corresponding differential gene analysis (i.e., within-line Leghorn or Fayoumi). **(C)** Pie charts showing the percentage of promoters or enhancers within the DERs of each histone modification. **(D)** Venn diagrams showing overlap of DERs across histone modifications. No overlap of H3K4me3 DERs was found for either comparison.

The promoters of all genes were assigned a chromatin state based on the criteria that first prioritize the overlap of specific promoter-enriched states, which if not applicable was then based on the most prevalent state overlap (further described in section “Materials and Methods”). The promoter state of each gene was compared between each group, following the comparisons completed for the gene expression data (Figure 4B). The first observation was that promoter states on the majority of DEGs did not change between two groups across all four comparisons. In addition, the majority of DEGs (81%) had active TSS (i.e., chromatin state 6), indicating that regulatory changes due to treatment or genetic line differences occur on constitutively active genes as opposed to regulation of poised or repressed genes (Figure 5B). Lastly, most promoter transitions between groups were moderate (i.e., within states of same activity) or to/from poised states (red/blue state to yellow state and *vice versa*) (Figure 4C).

The lack of or moderate changes seen in the promoter chromatin states of DEGs between groups may suggest that histone modification changes due to treatment or line differences are not dramatic enough to result in strong chromatin state transitions (i.e., active to silent state and *vice versa*), which are caused by differences in regions either being enriched versus not enriched between groups (i.e., peak calling). Instead, the regions that have assigned peaks in both groups being compared may still have significant changes in levels of enrichment. To explore this scenario, we examined the changes in histone modification enrichment levels at regulatory regions.

### Effects of Treatment and Genetic Line on Histone Modification Enrichment Levels

Histone enrichment levels have previously been shown to be correlated with biologically significant features such as enhancer activity, developmental state, and promoter conservation (Creyghton et al., 2010; Thakurela et al., 2015; Villar et al., 2015). Therefore, we explored the differences in the activity or priming of regulatory elements by measuring changes in enrichment levels of active (H3K4me3 and H3K27ac) or poised (H3K4me1) histone modifications, respectively. Enrichment levels were calculated by counting reads overlapping regulatory regions, which were determined from the chromatin states (see section “Materials and Methods”), for each histone modification. PCA plots of histone peak depth across samples showed clear clustering by line in all three histone modifications, while clustering by treatment can be observed in only H3K27ac and H3K4me1 (Supplementary Figure 6). The Spearman correlation was calculated between the fold changes of each histone modification enrichment level at promoters and gene expression of DEGs within each comparison (Supplementary Figure 7). The correlation with gene expression fold change across all comparisons showed that the greatest positive correlations with expression are with active histone modifications H3K4me3 and even more so with H3K27ac, which suggests that H3K27ac enrichment levels may be the most predictive of changes to gene expression levels among the four histone modification marks. Second, there was moderately positive correlation of expression with H3K4me1, and lastly, there was moderately negative correlation of expression with the repressive mark H3K27me3. On an interesting note, the greatest correlations occurred between histone marks H3K4me3 and H3K27ac, illustrating a biologically close relationship in regulation between the two histone modifications at promoters (Mikkelsen et al., 2007; Villar et al., 2015).

**FIGURE 6:**
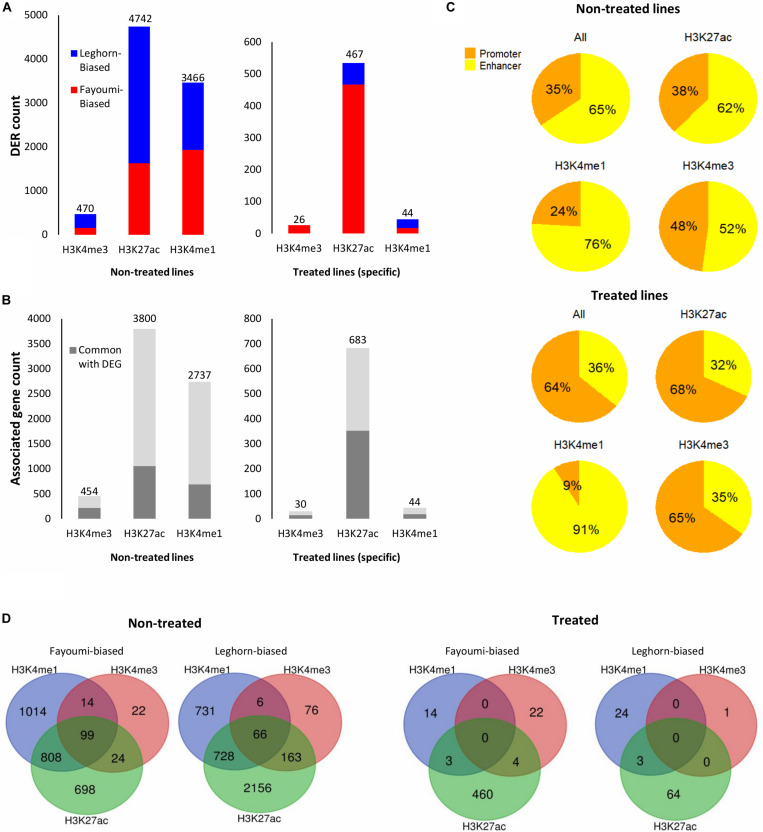
Summary of DER analysis for between-line comparisons. **(A)** Stacked bar plots showing the number of DERs with increased or decreased enrichment identified for each between-line comparison across all histone modifications. **(B)** Stacked bar plots showing the number of unique target genes associated with DERs and the number of associated genes that are in common with DEGs of the corresponding differential gene analysis (i.e., between non-treated lines or treated lines). **(C)** Pie charts showing the percentage of promoters or enhancers within the DERs of each histone modification. **(D)** Venn diagrams showing the overlapped DERs with similar line biases across histone modifications within each between-line comparison. DER, differentially enriched region; DEG, differentially expressed gene.

Differentially enriched regions were identified between each group using DESeq2 (FDR < 0.05). The numbers of DERs for within-line comparisons are summarized in Figure 6A. In both within-line comparisons, the greatest number of DERs appeared in H3K27ac (45 DERs in Fayoumi and 3,555 in Leghorn) and H3K4me1 (275 DERs in Fayoumi and 587 DER in Leghorn), whereas for H3K4me3 there were very few DERs detected (two DERs in Fayoumi and no DERs in Leghorn). This illustrates that H3K27ac and H3K4me1 were the most informative histone modifications among the three when exploring changes in regulatory regions due to treatment. The DER analysis mirrored the observations seen in the DEG analysis in that there appeared to be a larger effect of treatment on Leghorns as indicated by the greater number of DERs. Furthermore, the direction of change is similar, with the majority of the DERs having decreased enrichment due to treatment, reflecting the significant down-regulation of DEGs in Leghorn (Figure 5A). There was no complete overlap of DERs between the histone modifications, which illustrates the importance of examining multiple modifications as different indicators of chromatin changes during a response to treatment (Figure 5D). Lastly, prediction of target genes for regulatory elements has been determined in previous studies by proximity, which we have similarly implemented by assigning a target gene with the nearest TSS to a DER. We then compared the set of uniquely associated target genes with the set of DEGs from the same comparison group and found that the majority were not common between the two sets of genes (Figure 5B). There are several possible explanations for this: (1) the spatial dynamics of regulation results in the target gene not actually being the nearest gene, (2) the temporal dynamics of gene expression changes and histone modifications are not necessarily captured at the same time point, or (3) technical aspects in our assay or analysis. All statements described above were also observed in the between-line comparisons shown in the Figure 6, with the exception of one observation. In the between-line DERs, greater than 70% of H3K4me3 DERs overlapped with H3K27ac DERs, but without *vice versa* being true also (Figure 6D). These results suggest that between the two active histone modifications, the H3K27ac appears to be more informative by encompassing the majority of regions with H3K4me3 enrichment changes in addition to identifying significant numbers of other regulatory regions affected by treatment or line differences. Although there was moderate correlation of histone modification fold changes at the promoters of DEGs, there was high correlation of the fold changes between the active/poised histone modifications at DERs for both within-line and between-line comparisons, especially with H3K27ac compared to H3K4me3 or H3K3me1 (Supplementary Figure 8). This supports the hypothesis that active/poised histone modifications are concurrently regulated together and that changes in H3K27ac enrichment levels reflect its dynamic role in the activity of promoters and enhancers that has previously been shown in other studies (Creyghton et al., 2010; Thakurela et al., 2015).

We were interested in confirming the hypothesis of whether enhancers played a greater role than promoters in the differences seen during treatment or between lines as suggested above with the chromatin state transition analysis of the promoters of DEGs where there were small changes seen between groups (Figure 4B). In contrast to what we observed in the chromatin state promoter transition analysis, there were significant changes occurring in promoters where greater numbers of DERs were located in promoters in nearly all comparisons involving treated groups: 57% for within-line Fayoumi, 73% for within-line Leghorn, and 64% for between treated lines (Figures 5C, 6C). However, in the comparison between non-treated lines, we found that the majority of DERs (65%) were enhancers (Figure 6C). These observations suggest that inherent line differences are mainly regulated by enhancers, whereas response to treatment is mainly regulated by promoters.

### Functional Analysis of Regions With Histone Enrichment Level Changes

Using the associated target genes of DERs from within-line comparisons, the top 5 enriched GO terms and KEGG pathways are presented for each analysis. For DERs identified for H3K4me1, we found that both within-line analyses have very similar terms and pathways that relate to the immune system and, especially interestingly, are ones relevant to B-cell biology such as B-cell homeostasis, B cell receptor (BCR) signaling, and B-cell activation (Table 1). For H3K4me1, nearly all DERs had decreased enrichment with the treatment in both lines, which suggests possible down-regulation of B-cell function and development. For DERs identified for H3K27ac, there was only one enriched GO term in Fayoumi, which related to transcriptional regulation, but for Leghorns, there were many enriched GO terms and pathways that were similar to those found using the Leghorn DEGs such as protein regulation, cell division, and cell cycle (Table 1). Interestingly, the BCR pathway was enriched in Leghorn but not in Fayoumi, revealing a possible pathway that illustrates a significant difference in the effect of treatment on Leghorns versus Fayoumi.

**TABLE 1:** Treatment causes changes in H3K4me1 within regions that may function in B cell–related processes, and Leghorn has significant enrichment of processes related to bursal development, highlighting susceptible mechanisms to treatment compared to Fayoumi.

		Description	Count	−log10 *p*-value
**GO terms and KEGG pathways for genes associated with H3K4me1 DERs**				
Fayoumi	GO terms BP	B-cell homeostasis	4	2.88
		Positive regulation of GTPase activity	17	2.76
		Adaptive immune response	8	2.62
		B-cell receptor signaling pathway	5	2.36
		Positive regulation of transcription, DNA-templated	15	2.32
	KEGG pathway	Fc epsilon RI signaling pathway	6	2.71
		Fcγ R–mediated phagocytosis	6	2.31
		B-cell receptor signaling pathway	5	1.89
		HTLV-I infection	9	1.73
		Glycosaminoglycan biosynthesis—chondroitin sulfate/dermatan sulfate	3	1.55
Leghorn	GO terms BP	Adaptive immune response	18	7.00
		T-cell activation	11	6.88
		B-cell receptor signaling pathway	11	6.28
		B-cell activation	7	-0.20
		Transcription from RNA polymerase II promoter	28	3.92
	KEGG pathway	Fc epsilon RI signaling pathway	11	5.00
		T-cell receptor signaling pathway	12	4.22
		Fcγ R–mediated phagocytosis	11	4.18
		B-cell receptor signaling pathway	10	4.12
		Primary immunodeficiency	7	3.64
**GO terms and KEGG pathways for genes associated with H3K27ac DERs**				
Fayoumi	GO terms BP	Transcription from RNA polymerase II promoter	5	1.85
Leghorn	GO terms BP	Protein phosphorylation	88	10.15
		Positive regulation of transcription from RNA polymerase II promoter	151	9.25
		Cell division	71	9.20
		Positive regulation of transcription, DNA-templated	92	8.79
		Negative regulation of transcription from RNA polymerase II promoter	116	8.26
	KEGG pathway	B-cell receptor signaling pathway	30	10.95
		Fc gamma R-mediated phagocytosis	33	10.65
		Cell cycle	37	8.10
		Phosphatidylinositol signaling system	27	5.21
		Chronic myeloid leukemia	22	5.00

*GO terms and KEGG pathways were identified to be enriched in the target genes associated with the differentially enriched H3K4me1 and H3K27ac for each line. Significant processes were determined by p < 0.05 with gene counts of more than 2. The top 5 GO terms and KEGG pathways are shown for each line along with their gene count and -log10 p-value. BP, biological processes.*

We further explored enrichment of transcription factor motifs within H3K27ac DERs in order to identify any potential transcription factors with significant roles in the differences seen for our comparisons. Using only comparisons with larger numbers (>1,000) of DERs (TL vs. CL and CF vs. CL), the top 5 transcription factor motifs (similarity score ≥ 0.70) enriched in DERs for the non-treated-line and the within-line Leghorn comparison are presented (Supplementary Figure 9). In the non-treated-line comparison, the top 3 motifs belong to transcription factors with critical roles in B-cell development, highlighting possible differences between Leghorn and Fayoumi in their B-cell development in the bursa. The transcription factors enriched in Leghorn H3K27ac DERs have diverse roles in many cellular functions. ETS-like 1 (ELK1), Yin Yang 1 (YY1), Zinc finger X-chromosomal protein (ZFX), and NDT80 all function in processes that involve cell cycle and division, such proliferation, replication, and meiosis, while interferon regulatory factor 3 (IRF3) functions in type I interferon responses induced by viruses.

### Identifying Potential Functional Variants Between Genetic Lines

**FIGURE 7:**
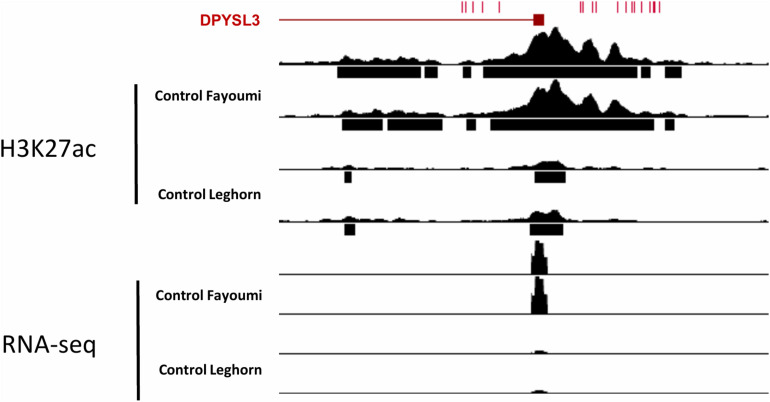
Genome browser track of the promoter of DPYSL3 with H3K27ac read pile-up and RNA-seq read pile-up of two biological replicates from control Fayoumi and control Leghorn. Red tick marks on top of the browser represent variants between the genetic lines.

To identify variants between the lines that potentially have functional consequences in genetic line differences associated with disease resistance, we identified homozygous variants (SNPs and small indels) between the lines that overlap coding and functional non-coding regions of the genome. After SNPs were filtered by quality, the SNPs were further filtered if they were heterozygous in either line, which allows more confidence that remaining SNPs are fixed among all individuals within each line. The total number of variants called within both lines was 5,808,195, and the total number of variants different between the lines was 3,521,496. Table 2 summarizes the total variants between lines that overlap genes and regulatory elements including DERs and DEGs identified from between-line comparisons. This reveals that there is a substantial number of candidate variants between lines that may be contributing to line differences, both inherent and in treatment response. Figure 7 shows an example of candidate variants overlapping the promoter of a DEG between the lines, dihydropyrimidinase-like 3 (*DPYSL3*) gene, which also overlap a DER for H3K27ac between the lines. This example illustrates the potential benefits of the integration of multiple genome-wide assays to identify potential causal variants affecting gene expression between these two lines.

**TABLE 2:** Variants between the genetic lines overlapping functional genomic regions.

	Total overlapped variants	% of total variants
Total within both lines	5,808,195	
Different between lines	3,521,496	60.63
Total regulatory regions	239,194	6.79
Total promoters	92,968	2.64
Total enhancers	146,226	4.15
Der between non-treated lines	83,740	2.38
DER between treated lines (specific)	10,596	0.30
Total exons	90,025	2.56
DEGs between lines (both treatment groups)	3,381	0.10

*Number of variants called within both genetic lines (versus reference galGal6 genome), number of variants different between the genetic lines, and number of variants different between the genetic lines that overlap total functional genomic regions and those different between the lines. Only homozygous variants within both lines were considered. The percentage of total variants is calculated from total variants within both lines.*

## Discussion

To capture changes due to simultaneous NDV infection and heat stress in both genes and regulatory elements, we performed differential analysis on the transcriptome, chromatin state transitions, and differential analysis on the histone modification enrichment levels for bursal tissue collected at 6 dpi from two genetically distinct inbred chicken lines, Fayoumi and Leghorn.

With treatment, differential gene analysis revealed a significant number of gene expression changes in the Leghorn line, but few in the Fayoumi line. Fayoumi showed enrichment in “extracellular matrix organization,” which is a biological process ubiquitous across all tissues and essential for proper development and tissue homeostasis (Bonnans et al., 2014). Leghorn displayed multiple enriched terms and pathways, but the ones most intriguing were those related to the housekeeping processes cell cycle and division. The significant down-regulation of these processes (Figure 1B) highlighted a potential detrimental effect of the treatment on Leghorns. Cell cycle and cell division are crucial aspects of cell proliferation, which is critical for bursa growth as B cells are rapidly dividing starting before hatch and up to the 4 weeks when the tissues were collected. Therefore, the down-regulation of these processes related to cell proliferation indicates that treatment may be hindering clonal division of B cells within the bursa of the treated birds, but more severely in the treated Leghorns. Previous studies in both chicken and mice suggest that this is more likely a result of the effects of heat stress rather than infection. Chickens that were subjected to heat stress had decreased lymphocyte count or antibody levels (Thaxton et al., 1968; Nathan et al., 1976; Prieto and Campo, 2010). These studies suggested that stress hormones, such as corticosterone or ACTH, which are increased during heat stress, may be the cause of these harmful effects on the humoral arm of the immune system. Indeed, mice under chronic stress from overexpressed corticotropin-releasing hormone from the hypothalamus had significantly decreased total B cells from both primary and secondary lymphoid organs and decreased antibody levels following immunization (Murray et al., 2001). This may explain a potential detrimental impact on B-cell development in bursa for the birds under chronic heat stress in our study.

Integration of chromatin state analysis with DEGs revealed an interesting finding. DEGs due to treatment or line differences had active chromatin state promoters in both groups within a comparison, which suggests that gene expression differences within any comparison mainly result from modulation of constitutively active genes. This phenomenon indicates that they likely play an important role in housekeeping functions. Indeed as described above, the enriched GO terms and KEGG pathways from DEGs identified in comparisons of treated versus non-treated groups for both lines were related to housekeeping processes.

The integration of multiple histone modifications to explore the chicken response to treatment provided a more comprehensive exploration into the biological significance of the effect of genetic variation than just analysis into the transcriptome alone. Both the functional analysis of the differential histone modifications of H3K4me1 and H3K27ac and the transcriptome analysis support the conclusion that treatment seems to affect cell proliferation of the bursa and more so in Leghorns than Fayoumis. As cell proliferation is a housekeeping process, the regulatory element bias toward histone modification enrichment changes in promoters during treatment is reasonable because transcription factors involved in housekeeping functions are enriched in binding sites that are proximal to TSS (Heidari et al., 2014). Moreover, the most significant enriched transcription factor motif in H3K27ac DERs for Leghorns (Supplementary Figure 8), which was 73% promoters (Figure 6C), was ELK1. Elk1 functions in ERK signaling pathway for positive regulation of the cell cycle and cell proliferation (Shin et al., 2011; Ducker et al., 2019) and has preferential bias for proximal-promoter binding when cobinding with GATA1 (Gerstein et al., 2012).

Exploring the histone modification differences between the two groups highlighted several interesting conclusions in the epigenetic changes due to treatment or line differences. Of the three active/poised histone modifications, the most informative appeared to be H3K27ac, which falls in line with what has previously been demonstrated (Creyghton et al., 2010; Villar et al., 2015; Gorkin et al., 2017; Fu et al., 2018). First, H3K27ac enrichment level fold changes had the highest correlation with fold changes of DEG and fold changes of enrichment levels of the other histone modifications, highlighting the predictive power of H3K27ac enrichment levels. Additionally, only H3K27ac enrichment level changes between the treated and non-treated groups were able to identify regulatory changes in biological processes and pathways uniquely within Leghorn, but not within Fayoumi. These pathways uniquely within Leghorn are very important for B-cell development and function, which may help explain Leghorns’ susceptibility to treatment. Lastly, the majority of DERs due to treatment occurred in promoters, whereas the majority of DERs due to line differences occurred in enhancers, illustrating a regulatory element bias for either the response to treatment or genetic line differences.

One of the more intriguing findings was that the enriched pathways and processes associated with H3K4me1 DERs between treatment groups for both lines and H3K27ac DERs between treatment groups in Leghorn were related to B-cell biology. The repeated appearance of the “B-cell receptor signaling pathway” enriched in DERs that were all nearly decreased with treatment (Table 1) suggests an important mechanism of B-cell development that is being repressed. This ties into the narrative of decreased B-cell proliferation suggested by our study and in previously mentioned chicken and mice studies with heat stress because loss of pre-BCR or BCR signaling arrests early B-cell development, thereby inhibiting maturation (Ferrari et al., 2007), or leads to induced death into mature B cells (Lam et al., 1997). This harmful effect of heat stress on B cells appeared in both lines, but it seems it was more exacerbated in Leghorns because of the greater changes seen in both transcriptome and histone modification analysis.

Leghorns have significantly lower levels of NDV-specific antibody titers compared to Fayoumis (Saelao et al., 2018b), which supports the notion that that Leghorns had more markedly decreased B-cell counts through inhibition of proliferation than Fayoumis. However, one caveat that must be considered is that B cells responsible for producing NDV-specific antibodies were most likely from peripheral tissues rather than from the bursa. This conclusion was drawn from several reasons. First is that a lentogenic strain of NDV will not systemically infect the bursa, which is supported by the fact that we did not detect viral transcripts from the bursa RNA-seq. Second, many B cells within the bursa are still maturing their BCR, the activation of which is necessary for differentiating B cells into secreting NDV-specific antibodies. Lastly, there is little evidence of the bursa acting as a peripheral lymphoid organ (Sorvari et al., 1975; Sorvari and Sorvari, 1978). Nonetheless, our results in the bursa provide an indication of what mechanisms may be affecting B cells throughout the body, including those responding to infection. Another possibility is that the impact of heat stress on bursa may have decreased the migration of B cells to peripheral tissues, thereby decreasing B-cell populations that are able to respond to infection. Our work provides some novel information on the impact of both heat stress and NDV on B-cell development in the bursa and laid the foundation of future studies into exploring the consequences of NDV infection under heat stress on peripheral B cells. Further studies by isolating B cells from multiple secondary lymphoid tissues during heat stress can further confirm our findings and examine our hypothesis regarding the impact of treatment on B-cell development and B-cell response to NDV infection.

The overall goal of our program is to genetically enhance resistance against heat stress and NDV infection in chickens. Therefore, we further explored genetic variants between the two lines that might be contributing to the resistance difference between the two genetic lines. Our analysis revealed a substantial number of variants between the lines that overlapped coding and functional non-coding regions of the genome. There were more variants that overlapped regulatory regions (6.79%) than exonic regions (2.59%), which suggest that the causal variants that affect line differences will most likely lie in functional non-coding regulatory regions as proposed by many GWAS. Indeed, a previous study exploring variants between the genetic lines observed that the majority of those variants were intergenic or intronic (Fleming et al., 2016). To further narrow down putative variants that are associated with line differences, we identified variants that overlapped all DEGs and DERs identified for between-line comparisons in both treated and non-treated groups. The listed putative variants generated from this study can be used to investigate disease resistance for further functional studies and highlights the advantage of integrating genome-wide multi-omics data that is currently appreciated in multiple projects for different species (ENCODE Project Consortium, Birney et al., 2007; Nègre et al., 2011; Roadmap Epigenomics Consortium, Kundaje et al., 2015), including livestock (Andersson et al., 2015).

Lastly, we speculated that there might be inherent differences between the two lines that allowed Fayoumi to be readily able to receive heat stress and NDV infection without significant genomic changes in response to treatment as shown by the small number of DEGs and DERs (Supplementary Figures 5A, 6A). RNA-seq analysis between the lines within treated birds showed that Fayoumi inherently had greater expression than Leghorns in genes involved in immune-related cell identity, suggesting they may have greater progression in bursal development (Figure 3). Furthermore, transcription factors such as PU.1, POU2F2, and IRF8 have very important roles in B-cell development and differentiation. These top enriched transcription factors in the H3K27ac DERs between non-treated Fayoumi and Leghorn suggest potential differences in binding activity between the lines, which implicate that B-cell development may vary between the lines. This is unsurprising given that bursal development can differ between lines (MiliĆEviĆ et al., 1986). Further studies collecting phenotypic data such as immune cell profiles and populations using flow cytometry across lymphoid tissues of the two lines are warranted to confirm this hypothesis.

## Conclusion

In summary, our study characterized the bursal response of chicken to NDV infection and heat stress and differences in responses between two different inbred chicken lines, Fayoumi and Leghorn. We found down-regulation of many genes within cellular processes such as cell division and cell cycle within Leghorn, suggesting a possible deleterious effect on bursal cell proliferation. The immune-related GO terms and pathways associated with regulatory regions with decreased enrichment levels in H3K27ac and H3K4me1 illuminated other potential mechanisms affected by heat stress and NDV infection. This potential harmful effect of stress on B cells induced by treatment may partly underlie why Leghorns are more susceptible to both heat stress and NDV infection. Further studies are needed to confirm the roles of these potentially significant mechanisms within bursa that play a role for resistance against NDV infection under heat stress. To our knowledge, our study is the first to identify genome-wide regulatory elements in the bursa tissue of chickens and therefore reveals novel insight into the chicken regulatory elements in bursa tissue and provides a great resource for other genomics-related chicken studies.

## Data Availability Statement

The datasets generated for this study can be found in online repositories. The names of the repository/repositories and accession number(s) can be found below: https://www.ebi.ac.uk/ena, PRJEB38599 and https://www.ebi.ac.uk/ena, PRJEB38600.

## Ethics Statement

The animal study was reviewed and approved by Institutional Animal Care and Use Committee at the University of California, Davis.

## Author Contributions

HZ, RG, and SL conceived study idea and developed experimental design. GC generated and analyzed data with guidance from CK. GC, YW, and PS collected the samples. YW assisted in experiments. GR provided technical advice on analysis. GC wrote the manuscript. All authors provided input into manuscript.

## Conflict of Interest

GR was employed by the company Zoetis. The remaining authors declare that the research was conducted in the absence of any commercial or financial relationships that could be construed as a potential conflict of interest.

## Footnotes

1 gip.ucdavis.edu
