# Supplementary material for: Integrated Transcriptome and Histone Modification Analysis Reveals NDV Infection Under Heat Stress Affects Bursa Development and Proliferation in Susceptible Chicken Line
Source: Front Genet. 2020 Sep 25;11:567812. doi: 10.3389/fgene.2020.567812 (PMC7545831; doi:10.3389/fgene.2020.567812)
Supplement: Supplementary file 1 [file Data_Sheet_1.PDF]

Supplementary Table 1: RNA-seq read alignment summary.
Raw reads were filtered by read quality, aligned to galGal6, and filtered by read mapping quality. Table summarizes total number of raw reads, aligned and filtered reads, and percentage of final read count.

Group	Sample ID	Batch	Raw Reads	Aligned and Filtered reads	% Final Reads
Control Fayoumi	1111	B	28,838,862	26,615,154	92.30%
Control Fayoumi	1126	A	31,400,000	26,763,852	85.20%
Control Fayoumi	1138	B	26,965,924	24,985,919	92.70%
Control Fayoumi	1159	A	36,221,494	30,893,377	85.30%
Control Leghorn	1008	A	34,891,983	29,438,846	84.40%
Control Leghorn	1020	A	27,700,841	23,630,504	85.30%
Control Leghorn	1044	B	26,071,042	24,036,335	92.20%
Control Leghorn	1052	B	26,691,886	24,676,374	92.40%
Treated Fayoumi	1107	A	37,928,760	32,492,516	85.70%
Treated Fayoumi	1114	B	31,121,401	28,733,849	92.30%
Treated Fayoumi	1120	A	34,859,303	29,005,466	83.20%
Treated Fayoumi	1132	B	26,748,264	24,732,844	92.50%
Treated Leghorn	1010	A	29,054,531	25,108,610	86.40%
Treated Leghorn	1039	A	41,892,529	35,461,511	84.60%
Treated Leghorn	1045	B	26,167,132	24,311,781	92.90%
Treated Leghorn	1050	B	27,143,444	24,901,140	91.70%

Supplementary Table 2: Peak calling and quality metrics summary.
A summary of the final read count after filtering and alignment, number of peaks called, the percentage of the genome covered by peaks, and quality metric scores including fraction of reads in peaks (FRIP) and Jensen-shannon distance (JSD). The number of peaks called for each group was determined by the combined peak calls of biological replicates within each group.

H3K4me3	Replicate	Final Reads	Peaks	Genome Coverage	FRIP	JSD	Group	Peaks
Control Fayoumi	1126	14492542	19697	2.2%	71%	0.69	Control Fayoumi	19556
Control Fayoumi	1159	18408651	19296	2.2%	56%	0.58	Control Leghorn	20368
Control Leghorn	1008	16195292	20931	2.3%	71%	0.69	Treated Fayoumi	10257
Control Leghorn	1020	15638996	19033	2.2%	74%	0.71	Treated Leghorn	18366
Treated Fayoumi	1107	18552688	13583	0.7%	8%	0.22		
Treated Fayoumi	1120	17257131	21050	2.2%	67%	0.66		
Treated Leghorn	1010	15108084	18046	2.0%	63%	0.64		
Treated Leghorn	1039	15188838	19801	2.1%	59%	0.61		
Median		15917144	19496.5	2.2%	65%	0.650		

H3K27ac	Replicate	Final Reads	Peaks	Coverage	FRIP	JSD	Group	Peaks
Control Fayoumi	1126	18798018	43980	3.6%	45%	0.5	Control Fayoumi	42509
Control Fayoumi	1159	19951339	41935	3.7%	44%	0.49	Control Leghorn	46746
Control Leghorn	1008	20925048	48085	3.8%	53%	0.56	Treated Fayoumi	32291
Control Leghorn	1020	19300463	45357	3.7%	54%	0.57	Treated Leghorn	46433
Treated Fayoumi	1107	37237111	33250	3.4%	28%	0.35		
Treated Fayoumi	1120	19526125	40702	3.3%	41%	0.47		
Treated Leghorn	1010	24054852	46707	3.7%	43%	0.48		
Treated Leghorn	1039	22337859	41568	2.9%	32%	0.4		
Median		20438193.5	42957.5	3.6%	44%	0.49		

H3K4me1	Replicate	Final Reads	Peaks	Coverage	FRIP	JSD	Group	Peaks
Control Fayoumi	1126	21690788	60581	5.1%	37%	0.4	Control Fayoumi	62555
Control Fayoumi	1159	23461873	74287	4.8%	39%	0.42	Control Leghorn	71746
Control Leghorn	1008	25154332	75173	5.1%	39%	0.44	Treated Fayoumi	38540
Control Leghorn	1020	26494210	74522	4.9%	36%	0.41	Treated Leghorn	65737
Treated Fayoumi	1107	51720903	44354	3.1%	15%	0.22		
Treated Fayoumi	1120	33063216	67666	4.2%	27%	0.32		
Treated Leghorn	1010	29449027	68184	3.8%	24%	0.31		
Treated Leghorn	1039	21028703	79526	4.1%	31%	0.4		
Median		25824271	71235.5	4.5%	34%	0.40		

H3K27me3	Replicate	Final Reads	Peaks	Coverage	FRIP	JSD	Group	Peaks
Control Fayoumi	1126	22658018	36073	7.2%	34%	0.36	Control Fayoumi	32814
Control Fayoumi	1159	25092629	38566	6.1%	29%	0.33	Control Leghorn	35464
Control Leghorn	1008	26231610	40492	6.7%	30%	0.32	Treated Fayoumi	13261
Control Leghorn	1020	28457039	37508	6.3%	28%	0.31	Treated Leghorn	30102
Treated Fayoumi	1107	38620070	15338	2.5%	9%	0.18		
Treated Fayoumi	1120	26307830	23818	3.9%	19%	0.26		
Treated Leghorn	1010	28445024	33898	5.4%	24%	0.29		
Treated Leghorn	1039	27478452	35473	5.8%	27%	0.31		
Median		26893141	35773	6.0%	28%	0.31		

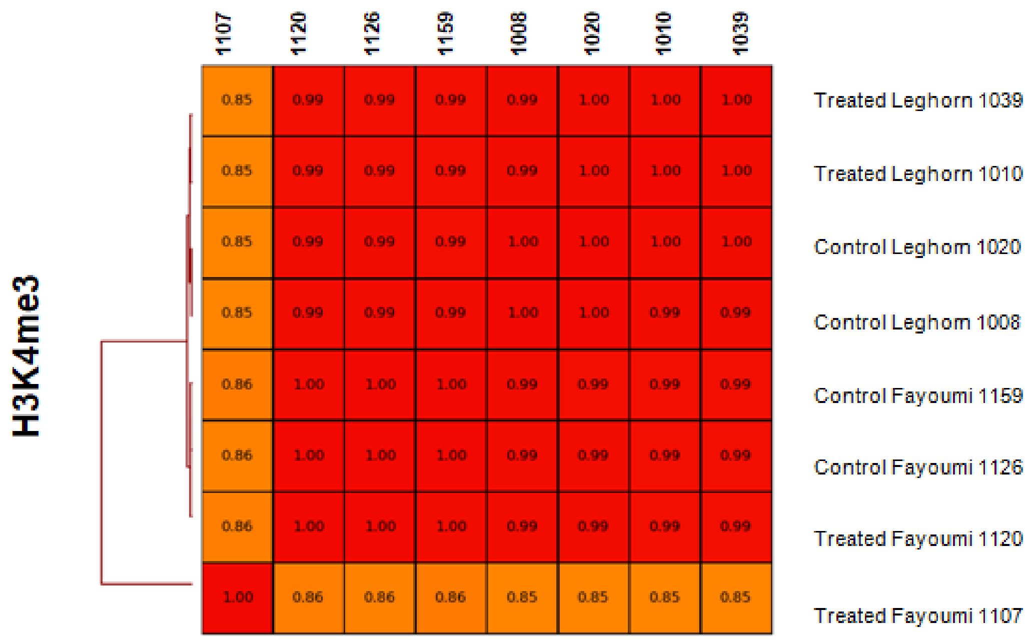
H3K4me3
1107 1120 1126 1159 1008 1020 1010 1039
0.85 0.99 0.99 0.99 0.99 1.00 1.00 1.00 Treated Leghorn 1039
0.85 0.99 0.99 0.99 0.99 1.00 1.00 1.00 Treated Leghorn 1010
0.85 0.99 0.99 0.99 1.00 1.00 1.00 1.00 Control Leghorn 1020
0.85 0.99 0.99 0.99 1.00 1.00 0.99 0.99 Control Leghorn 1008
0.86 1.00 1.00 1.00 0.99 0.99 0.99 0.99 Control Fayoumi 1159
0.86 1.00 1.00 1.00 0.99 0.99 0.99 0.99 Control Fayoumi 1126
0.86 1.00 1.00 1.00 0.99 0.99 0.99 0.99 Treated Fayoumi 1120
1.00 0.86 0.86 0.86 0.85 0.85 0.85 0.85 Treated Fayoumi 1107

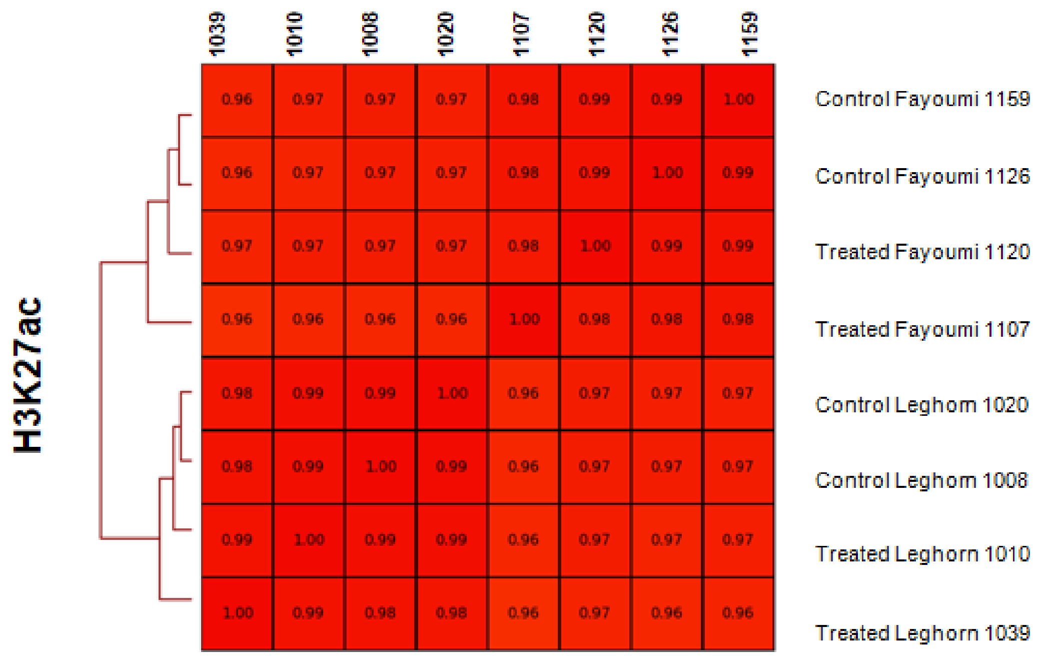
H3K27ac
1039 1010 1008 1020 1107 1120 1126 1159
0.96 0.97 0.97 0.97 0.98 0.99 0.99 1.00 Control Fayoumi 1159
0.96 0.97 0.97 0.97 0.98 0.99 1.00 0.99 Control Fayoumi 1126
0.97 0.97 0.97 0.97 0.98 1.00 0.99 0.99 Treated Fayoumi 1120
0.96 0.96 0.96 0.96 1.00 0.98 0.98 0.98 Treated Fayoumi 1107
0.98 0.99 0.99 1.00 0.96 0.97 0.97 0.97 Control Leghorn 1020
0.98 0.99 1.00 0.99 0.96 0.97 0.97 0.97 Control Leghorn 1008
0.99 1.00 0.99 0.99 0.96 0.97 0.97 0.97 Treated Leghorn 1010
1.00 0.99 0.98 0.98 0.96 0.97 0.96 0.96 Treated Leghorn 1039

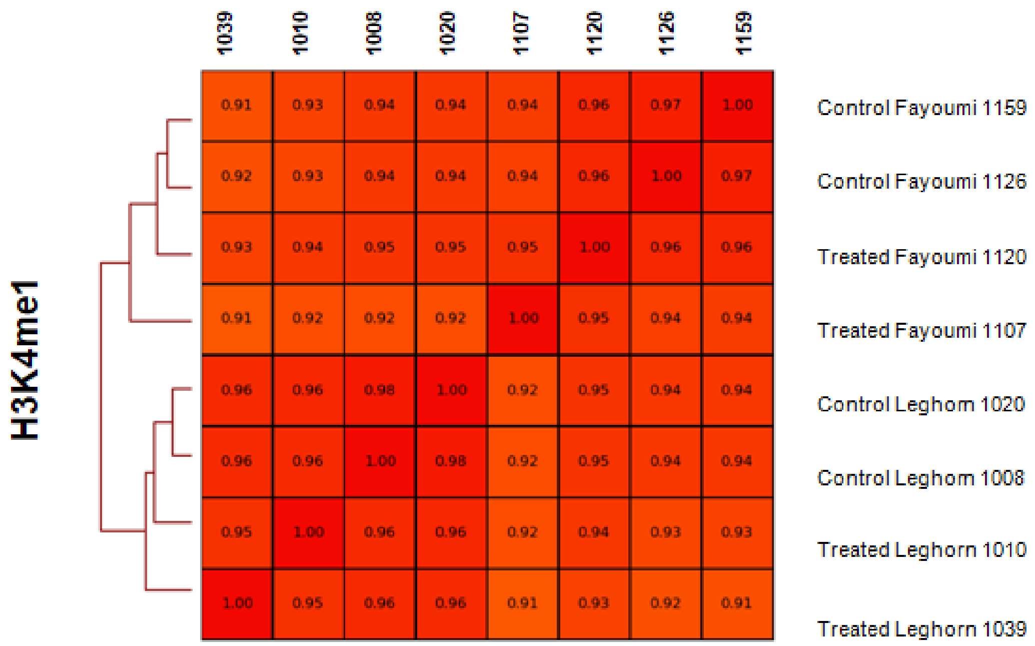

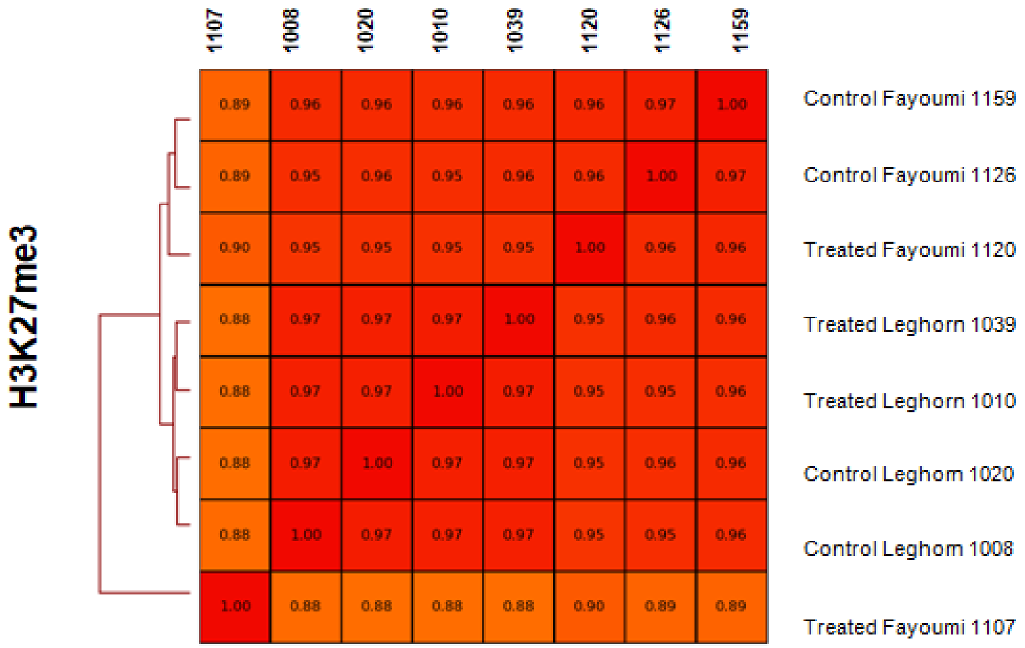

Supplementary Figure 1: Pearson correlation values between samples of histone modification ChIP-seq data. The genome was binned into regions of 1000bp where reads per kilobase million (RPKM) was calculated for each region and used in calculation correlation.

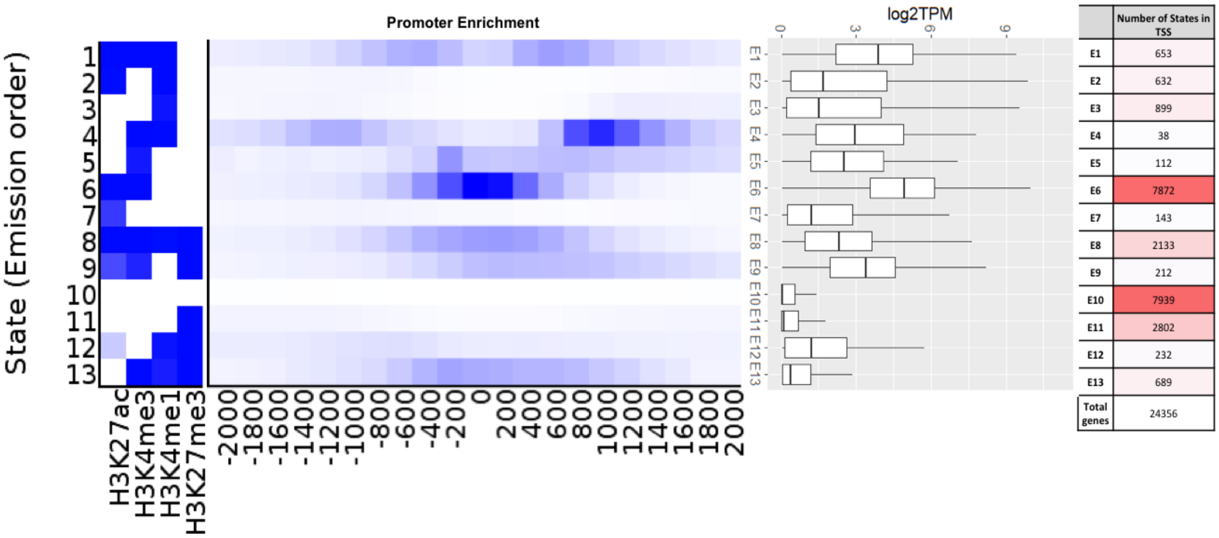

Supplementary Figure 2: Characterization of ChromHMM states around TSS. Promoter enrichment of each state +/- 2000bp around TSS. States 1, 4, 5, 6, 8, 9, and 13 appear to have moderate to high enrichment in the promoter region. Note that some states (6,8,9, and 13) are enriched directly on the TSS while others are flanking the TSS (1,4,5,12). Genes were assigned the state that overlapped its TSS and gene expression measured by TPM are shown in by boxplots. Average gene expression is higher for states containing H3K27ac while those containing H3K27me3 (in the absence of H3K27ac) have lower average gene expression. Data shown are from control Fayoumi group. TSS = transcription state site, TPM = transcripts per million.

	E1	E2	E3	E4	E5	E6	E8	E9	E12	E13
E1		41	26	95	72	92	48	33	22	30
E2	69		42	55	38	60	30	22	24	16
E3	93	91		96	71	87	50	38	47	34
E4	62	21	17		63	68	32	22	14	23
E5	29	9	8	39		39	19	21	8	14
E6	80	31	21	90	82		40	34	17	25
E8	25	9	7	26	25	25		68	38	77
E9	16	6	8	16	25	19	61		27	55
E12	24	15	14	23	21	21	79	62		86
E13	14	4	4	17	16	14	70	55	38	
No co-occurrence	0	6	44	0	6	1	0	12	24	0
Total number of states	10060	16992	36346	6575	4092	8748	5312	4799	10967	4817

Supplementary Figure 3: Co-occurrence of chromatin states. Heat map showing the percentage of a state's total region co-occurring with another state. Percentage is calculated from the number of regions where the row state and column state co-occur divided by the total number of regions of the column state. *No co-occurrence* denotes that a region has only that column state overlapped. States 7, 10, and 11 were removed from analysis for either being low/no signal or characterizing broad regions that do not identify regulatory elements such as promoters or enhancers.

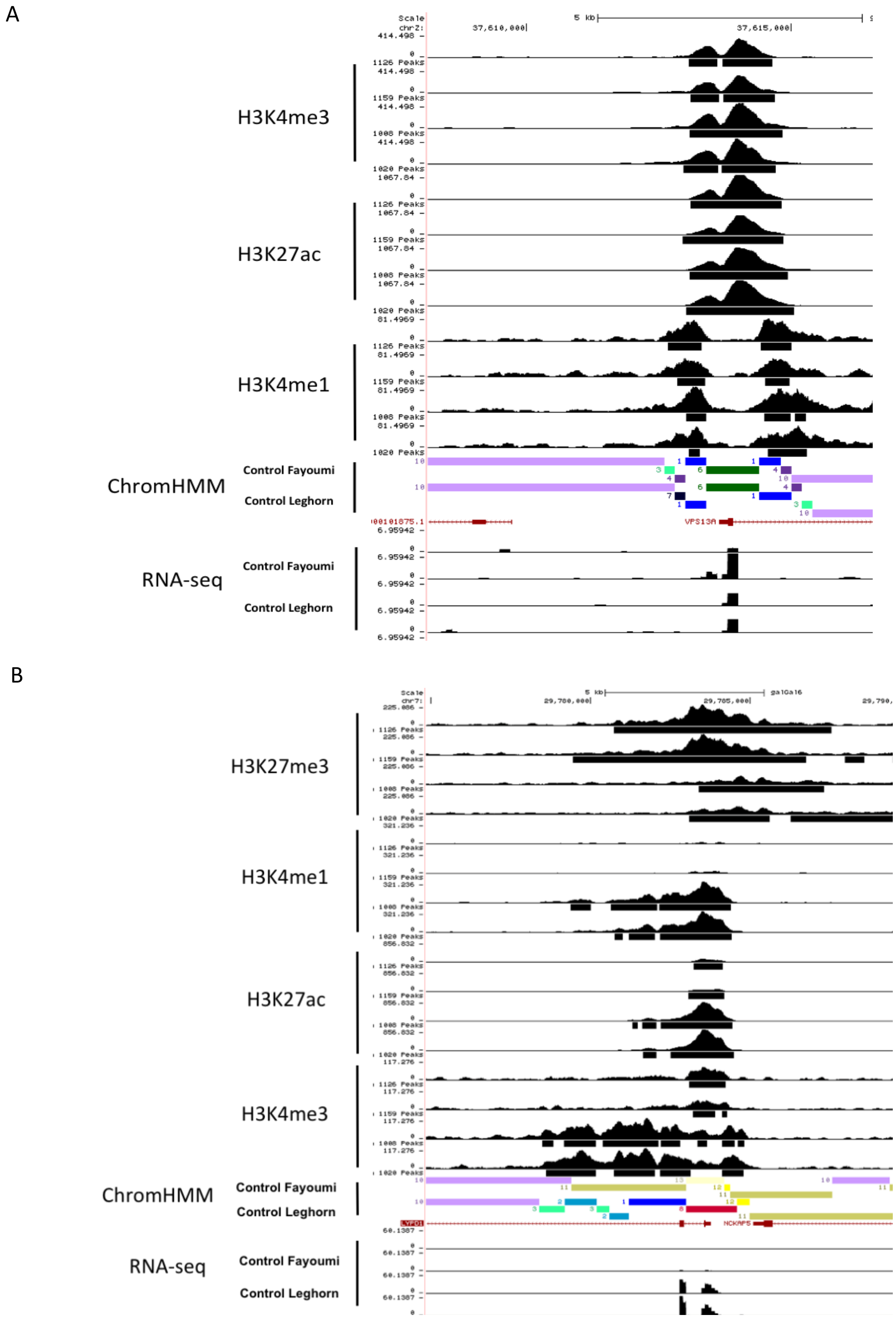

Supplementary Figure 4: Genome browser track of peak calls, read pile up, ChromHMM annotation, and RNA-seq read pile up for two samples in control Fayoumi and control Leghorn. (A) Track browser around promoter region of VPS13A gene. Note the similar and overlapping peak shapes between H3K27ac and H3K4me3 marks while H3K4me1 peaks are flanking those regions. This is reflected in the ChromHMM annotation where multiple states are resembling the relationship between the three histone marks that are characterizing the promoter region. The states in the browser do not match the colors in the table (which are colored according to activity) but are labelled accordingly with the state-model shown to the right. **(B)** Similar observations seen in 8A at the promoter region of this Leghorn-biased gene. Note that in control Leghorn ChromHMM annotation, there contains an active and poised state within the promoter region. This region also contains a broad peak region that generally characterizes the H3K27me3 mark. The histone modification bias towards Leghorn can be observed in the enrichment level differences, and interestingly length, in all marks where only H3K27ac peak calls can identify this bias.

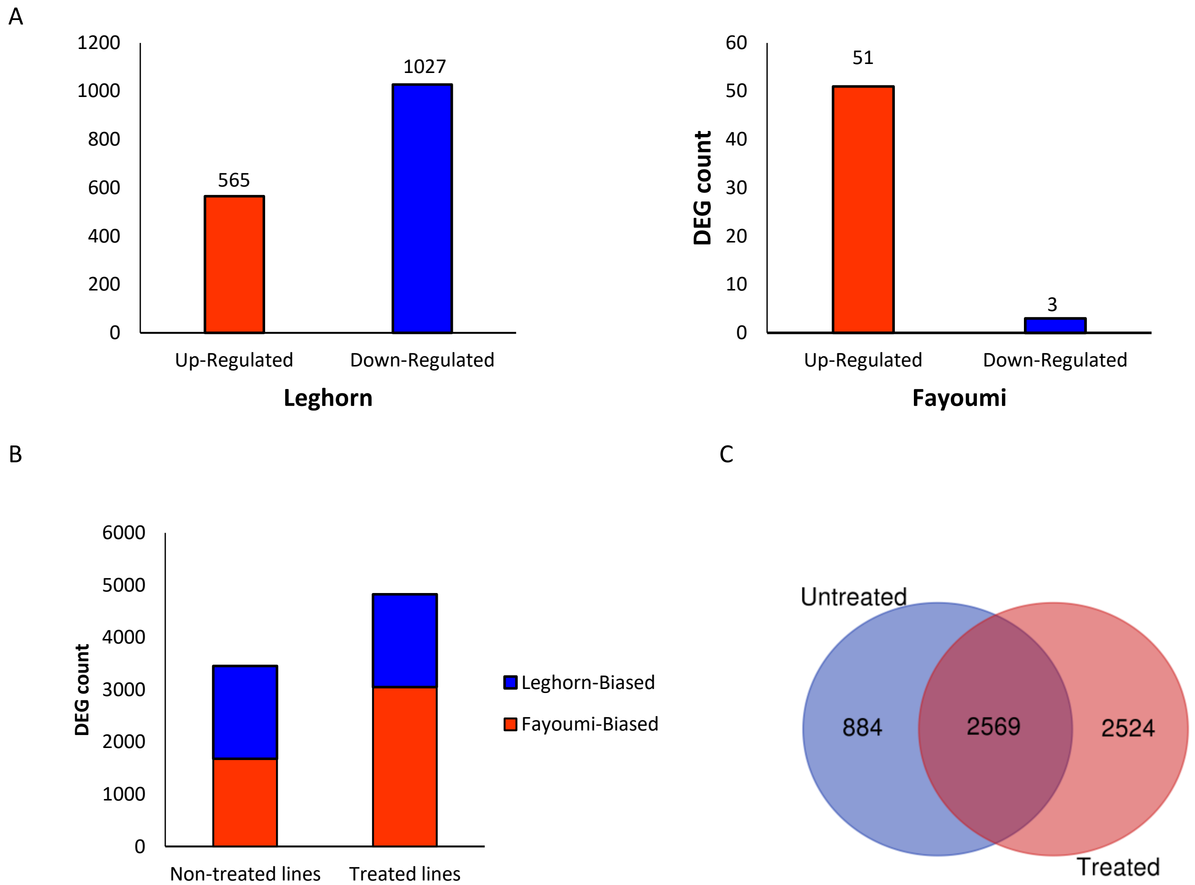

Supplementary Figure 5: Total count for differential gene analysis. (A) Count for up-regulated and down-regulated DEG within each line. **(B)** Count for Fayoumi-biased and Leghorn-biased DEGs for between-line comparisons. **(C)** Venn diagram showing the number of common and specific DEGs between the two comparisons.

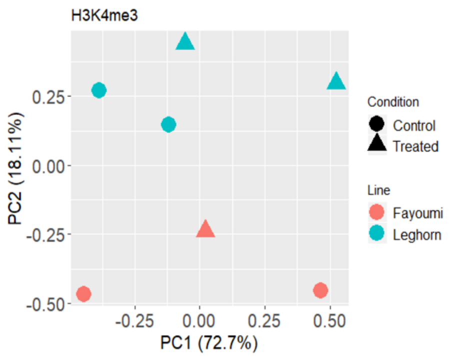

Total Regions	Promoters	Enhancers
45596	15856	32055

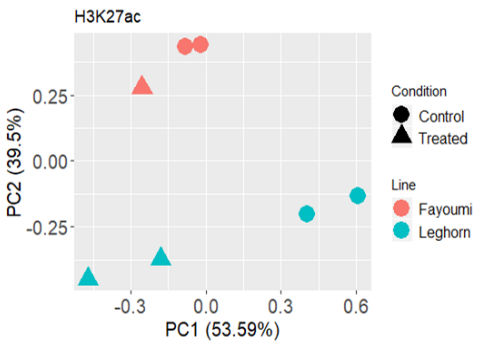

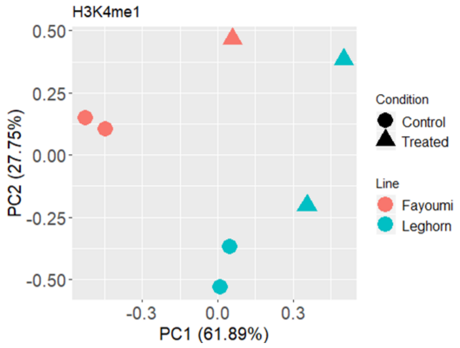

Supplementary Figure 6: Clustering by line and/or treatment visualized in PCA plots of histone enrichment levels over all regulatory regions. Normalized counts over regulatory regions were used for PCA and plots for PC1 by PC2 are shown above. Separation by line and/or treatment can be observed in all histone modifications.

DEG CF vs CL

	Gene	H3K4me3	H3K27ac	H3K4me1	H3K27me3
Gene	1				
H3K4me3	0.43	1			
H3K27ac	0.37	0.71	1		
H3K4me1	0.16	0.21	0.38	1	
H3K27me3	-0.31	-0.18	-0.063	0.17	1

DEG TF vs TL specific

	Gene	H3K4me3	H3K27ac	H3K4me1	H3K27me3
Gene	1				
H3K4me3	0.38	1			
H3K27ac	0.44	0.57	1		
H3K4me1	0.12	0.23	0.26	1	
H3K27me3	-0.04	-0.13	-0.08	-0.05	1

DEG TF vs CF

	Gene	H3K4me3	H3K27ac	H3K4me1	H3K27me3
Gene	1				
H3K4me3	0.21	1			
H3K27ac	0.3	0.53	1		
H3K4me1	-0.02	0.13	0.28	1	
H3K27me3	-0.0069	-0.17	-0.055	-0.24	1

DEG TL vs CL

	Gene	H3K4me3	H3K27ac	H3K4me1	H3K27me3
Gene	1				
H3K4me3	0.53	1			
H3K27ac	0.6	0.62	1		
H3K4me1	0.36	0.49	0.57	1	
H3K27me3	-0.43	-0.29	-0.43	-0.25	1

Supplementary Figure 7: Spearman Correlation of gene expression and histone modification changes at promoters of DEGs. Spearman correlation was calculated between fold changes of each histone modification enrichment level at promoters (4000bp centered on TSS) and gene expression of DEGs for each comparison. CF = Control Fayoumi, TF = Treated Fayoumi, CL = Control Leghorn, TL = Treated Leghorn.

A

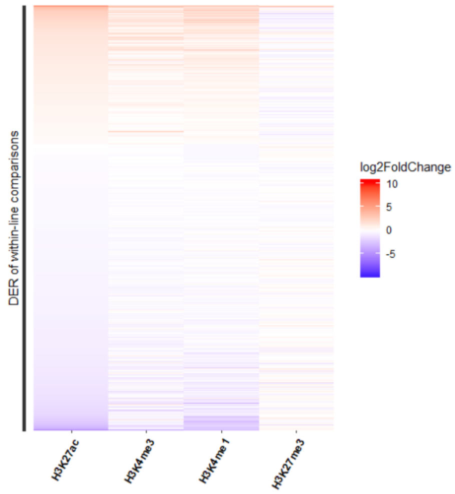

DERs of within-line comparisons

	H3K27ac	H3K4me3	H3K4me1	H3K27me3
**H3K27ac**	1			
**H3K4me3**	0.75	1		
**H3K4me1**	0.81	0.59	1	
**H3K27me3**	-0.25	-0.24	-0.14	1

B

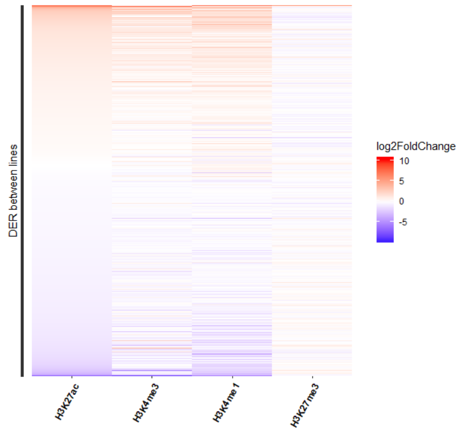

DERs of between-line comparisons

	H3K27ac	H3K4me3	H3K4me1	H3K27me3
**H3K27ac**	1			
**H3K4me3**	0.73	1		
**H3K4me1**	0.83	0.58	1	
**H3K27me3**	-0.2	-0.19	-0.06	1

Supplementary Figure 8: Changes to histone modification enrichment levels within DERs show high correlation with each other. (A) Heat map on the left shows log2 fold changes across all histone modifications for DERs identified in within-line comparisons and table on the left shows Spearman correlation between the log2 fold changes of DERs between the histone modifications for within-line comparisons. **(B)** Heat map on the left shows log2 fold changes across all histone modifications for DERs identified in between-line comparisons and table on the left shows Spearman correlation between the log2 fold changes of DERs between the histone modifications for between-line comparisons.

	DER CF vs CL			
	**Motif Description (TF/cell-type and method)**	**-log10pval**	**Similarity score**	**% of Targets**
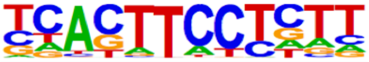	PU.1(ETS)/ThioMac-PU.1-ChIP-Seq	9.83	0.93	26.95
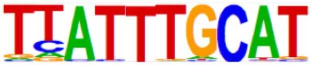	POU2F2/MA0507.1	6.23	0.92	23.85
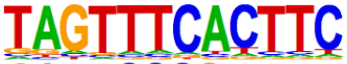	PU.1:IRF8(ETS:IRF)/pDC-Irf8-ChIP-Seq	5.96	0.88	5.13
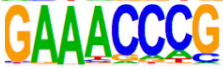	che-1/MA0260.1	5.34	0.84	56.91
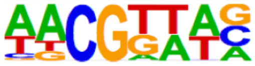	Pp_0229(RRM)/Physcomitrella_patens-RNCMPT00229-PBM	5.06	0.81	56.91

	DER TL vs CL			
	**Motif Description (TF/cell-type and method)**	**-log10pval**	**Similarity score**	**% of Targets**
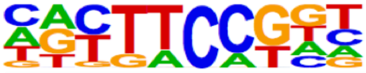	Elk1(ETS)/Hela-Elk1-ChIP-Seq	8.21	0.82	43.85
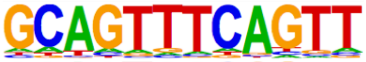	IRF3(IRF)/BMDM-Irf3-ChIP-Seq	5.82	0.79	9.11
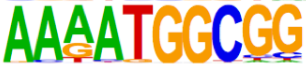	YY1/MA0095.2	5.67	0.91	7.36
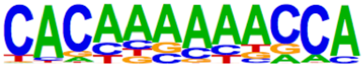	NDT80/MA0343.1	5.07	0.7	21.98
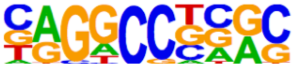	Zfx/MA0146.2	4.69	0.87	20.82

Supplementary Figure 9: Transcription factor motif discovery. HOMER was utilized to find enrichment of transcription factor motifs in H3K27ac DERs for non-treated line and within-line Leghorn comparison. Significance motifs were identified with p-value < 0.05 and similarity score of 0.7 or greater.
